# The St. Louis Dental Society

**Published:** 1893-03

**Authors:** 


### The St. Louis Dental Society.

The Annual Meeting of the St. Louis Dental Society was held at the office of Dr. J. B. Vernon, January 3, 1893. The following officers were elected for the ensuing year:

President, Dr. DeCourcy Lindsley; Vice-President, Dr. J. Warren Wick; Cor. Secretary, Dr. William Conrad; Rec. Secretary, Dr. J. G. Pfaff; Treasurer, Dr. Henry Fisher.

Committee on Publication: Drs. L. A. Young, P. H. Islœffel, and C. L. Pepperling.

Committee on Ethics: Drs. H. M. Baird, A. J. Prosser, and M. C. McNamara.

Committee on Membership: Drs. W. N. Morrison, C. L. Hickman and J. H. Spalding.

